# Family-based analysis of the contribution of rare and common genetic variants to school performance in schizophrenia

**DOI:** 10.1038/s41380-023-02013-2

**Published:** 2023-03-13

**Authors:** Alexandros Rammos, George Kirov, Leon Hubbard, James T. R. Walters, Peter Holmans, Michael J. Owen, Michael C. O’Donovan, Elliott Rees

**Affiliations:** Centre for Neuropsychiatric Genetics and Genomics, Division of Psychological Medicine and Clinical Neurosciences, Cardiff University, Cardiff, UK

**Keywords:** Schizophrenia, Genetics

## Abstract

Impaired cognition in schizophrenia is associated with worse functional outcomes. While genetic factors are known to contribute to variation in cognition in schizophrenia, few rare coding variants with strong effects have been identified, and the relative effects from de novo, inherited and non-transmitted alleles are unknown. We used array and exome sequencing data from 656 proband-parent trios to examine the contribution of common and rare variants to school performance, and by implication cognitive function, in schizophrenia. Parental transmission of common alleles contributing to higher educational attainment (*p* value = 0.00015; OR = 2.63) and intelligence (*p* value = 0.00009; OR = 2.80), but not to schizophrenia, were associated with higher proband school performance. No significant effects were seen for non-transmitted parental common alleles. Probands with lower school performance were enriched for damaging de novo coding variants in genes associated with developmental disorders (DD) (*p* value = 0.00026; OR = 11.6). Damaging, ultra-rare coding variants in DD genes that were transmitted or non-transmitted from parents, had no effects on school performance. Among probands with lower school performance, those with damaging de novo coding variants in DD genes had a higher rate of comorbid mild intellectual disability (*p* value = 0.0002; OR = 15.6). Overall, we provide evidence for rare and common genetic contributions to school performance in schizophrenia. The strong effects for damaging de novo coding variants in DD genes provide further evidence that cognitive impairment in schizophrenia has a shared aetiology with developmental disorders. Furthermore, we report no evidence in this sample that non-transmitted parental common alleles for cognitive traits contributed to school performance in schizophrenia via indirect effects on the environment.

## Introduction

Schizophrenia is a severe and often chronic psychiatric disorder characterised by a heterogeneous psychopathology [1]. Impaired cognitive ability is a key feature as it is associated with worse functional outcomes [2]. There is strong evidence that cognitive impairment in people with schizophrenia is present premorbidly [3], with childhood impairments across cognitive domains observed more commonly in those who subsequently develop schizophrenia [4]. Moreover, the rate of intellectual disability (ID) in people with schizophrenia is greater than the rate of ID in the general population [5]. There is mixed evidence for association between lower school performance and schizophrenia, although the largest population study on this subject reported lower school performance as a risk factor for schizophrenia [6]. There is also evidence that some of the cognitive deficits associated with schizophrenia occur after the onset of the disorder [7–9]. A lack of treatment options to improve cognitive function underscores the importance of understanding the biological basis of cognitive impairment in schizophrenia.

Schizophrenia and cognitive ability in the general population are highly heritable and have partly overlapping polygenic architectures [10–13]. Thus, common genetic liability to schizophrenia is negatively correlated with that for higher IQ (with coefficients ranging between −0.2 and −0.4) [11]. Rare copy number variants (CNVs) that increase risk for schizophrenia are associated with poorer cognitive function in the general population [14, 15]. Genes associated with rare coding variants in early-onset developmental disorders (DDs), including ID, are also enriched for de novo coding variants in people with schizophrenia [16].

There is evidence that both common and rare variants contribute to cognitive ability in people with schizophrenia. Common variants associated with higher IQ or with higher educational attainment (EA) in the general population are associated with higher cognitive function and EA in schizophrenia [11, 17–20]. However, while common genetic liability for schizophrenia is associated with poorer cognition and EA in people without schizophrenia, its effects on cognition and EA in people with schizophrenia remain unclear [17, 20]. In studies of rare variants, CNVs that confer risk for schizophrenia, and CNVs that affect genes with evidence for selective constraint against protein-truncating variants (PTVs), known as loss of function intolerant (LoFi) genes, are associated with poorer premorbid and current cognitive function, but not with EA, in schizophrenia [17, 20, 21]. Rare coding variants in LoFi genes, and rare coding variants in genes associated with DDs, are enriched in people with schizophrenia and comorbid ID compared to schizophrenia without ID [22], while also contributing to deficits in premorbid cognition and current cognition in schizophrenia [23]. Finally, population studies have shown rare coding variants in LoFi genes are associated with lower EA in people with schizophrenia [17]. Common genetic liability for IQ and EA, rare CNVs, and rare coding variants have, in part, independent effects on cognition in schizophrenia [17, 23]. However, the combined effects from these common and rare variants only explain around 10% and 6.2% of the variance in premorbid and current cognition in schizophrenia, respectively [23].

Studies of educational outcomes in the general population have consistently shown that parental variants transmitted to their offspring have direct effects on offspring EA [24]. Parental variants that are not transmitted to offspring also have indirect effects on offspring EA, albeit weaker than the direct effects of transmitted variants, through their contribution to the child’s environment [25]. This phenomenon is known as genetic nurture [24, 25]. The possible role of genetic nurture in EA in people with schizophrenia has not yet been studied.

Here, in a Bulgarian schizophrenia proband-parent trio sample, we investigated the contribution of common and rare variants to school performance, and by implication cognitive function, as measured by final school grades obtained around age eighteen. By using a trio study design, we were able to separate out the effects coming from de novo, transmitted, and non-transmitted alleles in schizophrenia, which, to our knowledge, has not been done before. Moreover, we analysed non-transmitted alleles to perform the first investigation of genetic nurture effects on school performance in schizophrenia.

For common variants, we hypothesised the strongest effects on school performance would come from transmitted alleles defined from Genome Wide Association Studies (GWAS) of EA and intelligence, given recent findings from case-control and population cohort studies [17, 20]. In view of the evidence for genetic nurture effects on educational outcomes in the general population [24, 25], we also hypothesised that non-transmitted alleles defined from GWAS of EA and intelligence would have indirect effects on school performance in schizophrenia, albeit weaker than the direct effects observed for transmitted alleles. For rare variants, we hypothesised that damaging coding variants and CNVs affecting LoFi genes and genes associated with DDs would be associated with lower school performance in schizophrenia, with the strongest effects coming from de novo variants, followed by ultra-rare transmitted variants, given evidence that newly arising mutations are enriched for more deleterious types of mutation [26].

## Methods

### Sample description

The sample was made up of 699 Bulgarian parent-proband trios who had school performance phenotype data available, of which 662 had exome sequencing data, 693 had array genotype data, and 656 had both exome sequencing and array genotype data. Recruitment took place between 1999 and 2004 in several psychiatric hospitals in Bulgaria. Ethical Committee approval was obtained from each of these hospitals (see Supplemental Material for full information). All probands and all parents received an Information Sheet and signed Informed Consent Forms. All probands had been previously hospitalised and met DSM-IV criteria for either schizophrenia (*N* = 594) or schizoaffective disorder (*N* = 105). All individuals attended mainstream schools, which, in Bulgaria, excludes individuals with evidence of severe or moderate ID. Genetic sex was available for all subjects (351 male and 348 female probands). More details on the sample and the process of recruitment can be found in Kirov et al. [27]. Although a subset of the current sample has previously been used to show a higher exome-wide rate of de novo PTVs in probands with lower school performance [28], the current study exploits recent advances in understanding the nature of selective constraint against rare coding variants [29], as well as the identification of specific developmental disorder risk genes [30], to prioritise rare variants that are more likely to impact school performance in schizophrenia.

### Cognitive phenotype data

School performance was measured using the final rounded grade awarded at the end of high school. The grades ranged between 6 (the best possible grade) and 3 (the lowest passing grade). In the Bulgarian high school system, a grade of 2 is considered a failing grade, and students who fail are required to repeat a school year. The probands included in our study were required to have completed mainstream school, and therefore all probands have a school grade of at least 3. Given some probands with a grade of 3 will have repeated a school year, those with a grade of 3 will have a range of abilities. The distribution of school grades within our sample can be seen in Supplemental Fig. S1. For our analysis of school performance, we combined the top two grades (6–5) and the bottom 2 grades (3–4), to create a binary measure of school performance, which is in line with previous work that has been conducted in this sample [28].

### Genetic data and quality control

#### Array genotype data

All trios were genotyped on Affymetrix 6.0 arrays at the Broad Institute of Harvard and Massachusetts Institute of Technology as described [27].

#### Copy number variant calls

A detailed description of how de novo CNVs were identified in this sample is provided elsewhere [27]. Briefly, CNV calls were excluded if they had a frequency >1% in the sample, were covered by <15 probes, overlapped segmental duplications by >50% of their length, or were smaller than 15KB. All de novo CNVs included in the current study were validated using custom Agilent arrays [27]. For transmitted and non-transmitted CNVs, we applied a more stringent CNV size threshold that excluded CNVs <100 KB as these were not independently validated [27, 31]. Transmitted CNVs were defined as those observed in a proband that had any overlap with a CNV observed in at least one of their parents. Non-transmitted CNVs were those observed in a parent but not their child.

#### Common variant calls

SNP genotype data were imputed using the HRC reference panel (v1.1) [32]. After excluding variants with low imputation quality (info score < 0.8), the genotype dataset consisted of 4 846 189 SNPs. SNPs were then excluded on the basis of a genotyping rate < 0.99, Hardy–Weinberg disequilibrium (*P* < 1 × 10^–6^), or a minor allele frequency less than 1%, retaining 1 316 770 SNPs. No individuals were removed for missing genotype data, defined as a genotype call rate < 0.99; in addition, no trios or SNPs were removed due to mendelian error rates (i.e., all SNPs were consistent with Mendelian inheritance in > 90% of trios, and within trios > 90% of all SNPs were consistent with Mendelian inheritance). Subsequently, we used principal component analysis (PCA) on the remaining 78,653 SNPs, after pruning using a sliding window size of 50 KB, a step size of 5 variants and a R^2^ threshold of 0.1 with Plink [33], to identify population structure in the current sample. Using the first two principal components, we were able to identify ancestry outliers within our cohort (see Supplemental Fig. S2). We included all trios in our primary analysis and controlled for population structure by adjusting for the first 10 principal components in all regression analyses.

#### Exome sequencing data

Exome sequencing data were generated as described [28, 34]. Briefly, 617 proband-parent trios were sequenced using either Agilent hybrid capture or Nimblegen array-based capture, with all members of a trio sequenced using the same capture, followed by paired-end sequencing on Illumina HiSeq sequencers. Raw sequence data were reprocessed as part of the SCHEMA consortium using the BWA-Genome Analysis ToolKit (GATK) best practice guidelines [35]. An additional 72 proband-parent trios were sequenced using the Nextera DNA Exome capture kit on the Illumina HiSeq platform [34]. Raw sequence from these trios were again processed using the BWA-GATK best practice guidelines [34].

#### Coding variant calls

To obtain a high-quality call set for autosomal transmitted and non-transmitted coding variants, all members of a trio where at least one individual was a carrier were required to pass the following genotype filters: sequencing depth ≥ 10X, genotype quality score ≥ 30, alternative allele balance ≤0.8 and ≥0.2 for heterozygous genotypes, ≥0.9 for homozygous genotypes of the non-reference allele and ≤0.1 for homozygous genotypes of the reference allele. De novo coding variants were obtained from our earlier published studies [28, 34].

#### Coding variant annotations

Coding variants were annotated using the Ensemble Variant Effect Predictor (version 96) [36]. PTVs included stop-gain, frameshift, and splice donor/acceptor variants. Missense variants were annotated with their “Missense badness, Polyphen-2, constraint” (MPC) score. CADD scores were also used to prioritise deleterious PTVs and missense variants. We defined damaging PTVs as frameshift variants, stop-gain variants with a CADD score ≥ 20, and splice donor/acceptor variants with a CADD score ≥ 20. CADD scores were used to prioritise damaging stop-gain variants and splice donor/acceptor variants, given stop-gain variants with higher CADD scores are more strongly enriched in developmental disorders [37]. We defined damaging missense variants as those with an MPC score ≥ 1 and a CADD score ≥ 20, since missense variants prioritised with both CADD scores and regional measures of missense constraint are more strongly enriched in developmental disorders than variants prioritised with CADD scores or measures of missense constraint alone [37]. We term damaging PTVs and damaging missense variants collectively as “damaging coding variants”. In the analysis of transmitted and non-transmitted coding variants, we focussed on ultra-rare variants, defined as variants observed once among all Bulgarian parents and absent among 60,146 individuals in the gnomAD control dataset (gnomAD v2.1.1) [29].

### Quantifying genetic liability for common and rare variants

#### Common variants

Schizophrenia polygenic risk scores (PRS) were generated using a custom version of the most recent GWAS of schizophrenia [38], which excluded the Bulgarian trio sample. We also generated PRS based on GWAS studies of EA [39] and intelligence [13]. We harmonised the summary statistics with the SNPs available in our sample using an extension of an in-house pipeline [32]. SNPs were clumped within a physical distance of 250 KB, with a linkage disequilibrium (LD) threshold *R*^2^ of 0.2 using the PRSice software [40], before scoring. Scores were generated across three training data *p* value thresholds (*p* < 0.001, 0.05, 0.5). The primary analysis of PRS used scores generated with a *p*-threshold of 0.05, as this explains the most case-control variance in schizophrenia [38]. We included the other *p* value thresholds as a sensitivity analysis to ensure robustness to thresholding.

Non-transmitted alleles at each SNP were used to create a pseudo-control for each proband-parent trio using the Plink command -tucc [33]. From these alleles, we generated a non-transmitted PRS (nt-PRS) using PRSice for each trio. The nt-PRS was used to quantify the effects of genetic nurture, that is the indirect effects of non-transmitted parental alleles on the offspring [25].

Polygenic transmission disequilibrium test (pTDT) scores were generated to evaluate over or under transmission of polygenic loading [34, 41]. pTDT scores were generated for each trio by subtracting the mean parental PRS from the proband PRS. These were then standardised by dividing the resultant value by the mean parental PRS standard deviation.

#### Rare variants

We tested six classes of rare genetic variation for association with lower school performance: (1) Damaging de novo coding variants; (2) Damaging transmitted coding variants; (3) Damaging non-transmitted coding variants; (4) De novo CNVs; (5) Transmitted CNVs and (6) Non-transmitted CNVs.

For each proband and class of rare genetic variation, we quantified the number of variants in the following gene-sets: (1) LoFi genes (*n* = 3063) with gnomAD pLi scores > 0.9 and (2) DD genes (*n* = 726), defined as confirmed or probable developmental disorder genes in the DDG2P database that are associated with a monoallelic mode of inheritance (i.e. variants identified on one allele in all or the vast majority of individuals with a specific developmental disorder) [30]. We defined CNVs in these sets as those that overlapped any exonic region for at least one gene that belonged to the set. In total, we tested 12 different rare variant mutation categories (6 mutation classes × 2 gene sets).

### Study design and multiple testing correction

Our primary analysis of common and rare variants consisted of 18 univariable logistic regression tests, where the outcome was a binary measure of high or low school performance. These tests included 6 categories of common variant (transmitted and non-transmitted PRS for schizophrenia, EA and intelligence) and 12 categories of rare variants (described above). Therefore, statistical significance in our primary analysis was determined as *P* < 0.0027 (0.05/18). Common variant pTDT analyses and rare variant TDT analyses were performed as secondary tests to seek additional support for our primary findings.

### Statistical analysis

#### Univariable analysis

Firth’s logistic regression [42] was used to test for association between each category of rare variant and school performance. Binomial logistic regression was used to test for association between standardised PRS and school performance. Principal components 1–10 and sex were included as covariates in all univariable regression analyses.

#### Multivariable analysis

Firth’s logistic regression [42] was used to test the independent effects of different classes of common and rare variants within the same model. In the multivariable model, we included genetic components that were significantly associated with school performance in the individual univariable analyses. The multivariable model was defined as:

$$\log \left[ {\frac{{P\left( {{{{{{\rm{Binary}}}}}}\,{{{{{\rm{Score}}}}}}} \right)}}{{1 - P\left( {{{{{{\rm{Binary}}}}}}\,{{{{{\rm{Score}}}}}}} \right)}}} \right] = 	 \;\alpha + \beta _1\left( {{{{{\rm{PC1}}}}}} \right) + \cdot \cdot \cdot + \beta _{10}\left( {{{{{\rm{PC10}}}}}} \right) + \beta _{11}\left( {{{{{\rm{Sex}}}}}} \right) \\ 	 + \beta _{12}\left( {{{{{{\rm{Genetic}}}}}}\,{{{{{\rm{Factor1}}}}}}} \right) + \cdot \cdot \cdot + \beta _{n + 11}\left( {{{{{{\rm{Genetic}}}}}}\,{{{{{\rm{Factor}}}}}}\,n} \right)$$

Nested binomial logistic regression models were used to calculate the variance explained by each individual genetic component, by calculating the difference in pseudo-R squared (Nagelkerke’s) between the full model and a reduced model that excludes the genetic factor whose contribution we want to measure.

#### Rare variant TDT analysis

Deviations from the expected chance transmission rate for ultra-rare coding variants and rare CNVs were evaluated using an asymptotic chi-square test with one degree of freedom.

#### Exact binomial proportion confidence interval

The exact binomial proportion confidence interval function in R was used to calculate 95% confidence intervals for the proportion of people carrying a specific class of rare variant who have lower school scores.

#### PRS power calculation

To estimate the power that our polygenic risk score analysis needed to detect association with school performance with an alpha of 0.05, two independent variables (PRS, V2) with normal distributions (0,1) were first generated to create a quantitative liability *L*, which was defined as $L = ( {\sqrt {R_{{{{{\rm{PRS}}}}}}^2} } )\;{{{{{\rm{PRS}}}}}} + ( {\sqrt {1 - R_{{{{{\rm{PRS}}}}}}^2} } )\;{{{{\rm{V}}}}}2$. Since there were approximately equal numbers of individuals with higher and lower school performance in the sample, polygenic scores were then assigned to individuals with lower school performance if *L* < 0, or individuals with higher school performance if *L* > 0, until a pre-defined sample size was reached. The two samples were then compared using a *t*-test and the above process was repeated 10,000 times to derive exact power estimates, with power defined as the proportion of simulated datasets where the *t*-test had nominal significance (*p* value < 0.05).

## Results

### Common variant analysis

**Fig. 1:**
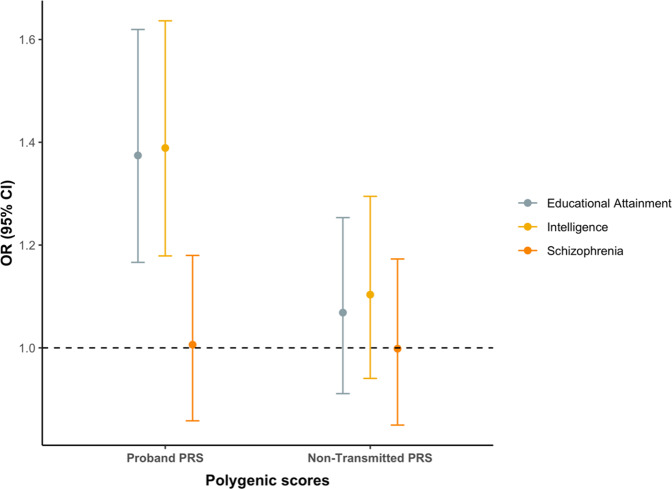
Univariable analysis of polygenic risk scores and school performance. Odd ratios shown are for higher school performance relative to lower school performance, for one standard deviation change in PRS. Logistic regression models were adjusted for sex and 10 principal components.

PRS for EA and intelligence were significantly associated with higher school performance after correction for multiple testing (EA PRS: OR = 1.37, uncorrected *p* value = 1.5 × 10^–4^; Intelligence PRS: OR = 1.39, uncorrected *p* value = 8.6 × 10^–5^), while PRS for schizophrenia was not (uncorrected *p* value = 0.94; Fig. 1). Comparable results were observed for PRS generated from additional P value thresholds in the source GWAS (Supplemental Table S1).

In the secondary pTDT analysis, there was evidence for greater over-transmission of PRS for EA and intelligence, but not for schizophrenia, among probands with higher school performance compared with those with lower school performance (Supplemental Table S2).

The parents of probands with higher school performance had significantly higher PRS for EA and intelligence than the parents of probands with lower school performance (Supplemental Table S3, Supplemental Fig. S3); however, there were no significant differences between the two groups in the non-transmitted PRS for EA, intelligence or schizophrenia (Fig. 1, Supplemental Table S1). We estimate that our study had ~80% power at an alpha of 0.05 to detect a non-transmitted PRS association that explained ~1.8% of the variance in school performance.

### Rare variant analysis

A total of 66 damaging de novo coding variants were identified within LoFi genes in our sample. These were enriched in the probands with lower school performance (OR = 2.07; *p* value = 4.5 × 10^–3^; Table 1), although this association would not survive Bonferroni correction for 18 tests. Of the damaging de novo coding variants, 13 were identified in DD genes, of which 12 were in individuals with lower school performance (OR = 11.6; *p* value = 2.6 × 10^–4^; Table 1), an association that surpassed the correction threshold. The proportion of people carrying damaging de novo coding variants in LoFi genes and DD genes who had lower school performance was 60.6% (47.8–72.4%) and 92.3% (64–99.8%), respectively.

**Table 1:** Univariable analysis of rare genetic variation and school performance, controlling for sex and 10 principal components.

		LoFi genes				DD genes			
Variant type	Inheritance type	OR (95% CIs)	*p* value	Average number of variants carried by probands (*n* variants)		OR (95% CIs)	*p* value	Average number of variants carried by probands (*n* variants)	
				Lower school scores	Higher school scores			Lower school scores	Higher school scores
Damaging coding variants	De Novo	2.07 (1.25, 3.5)	0.0045	0.14 (40)	0.070 (26)	11.6 (2.74, 107)	0.00026	0.041 (12)	0.0027 (1)
	Transmitted	1.01 (0.92, 1.12)	0.79	1.53 (447)	1.58 (582)	0.94 (0.75, 1.18)	0.59	0.39 (114)	0.41 (152)
	Non-transmitted	0.98 (0.89, 1.09)	0.76	1.43 (419)	1.50 (553)	0.83 (0.64, 1.07)	0.14	0.32 (94)	0.41 (150)
CNVs	De Novo	1.51 (0.61, 3.8)	0.37	0.035 (11)	0.023 (9)	0.48 (0.05, 2.7)	0.42	0.003 (1)	0.01 (4)
	Transmitted	1.19 (0.78, 1.82)	0.41	0.13 (40)	0.12 (46)	1.68 (0.62, 4.56)	0.31	0.026 (8)	0.021 (8)
	Non-Transmitted	0.86 (0.56, 1.3)	0.47	0.13 (39)	0.14 (53)	1.47 (0.64, 3.41)	0.36	0.039 (12)	0.029 (11)

Probands with lower and higher school scores are coded as 1 and 0 in the Firth’s logistic regression models, respectively. The sequencing analysis of damaging coding variants involved 293 and 369 probands with lower and higher school scores, respectively. The array-based analysis of CNVs involved 310 and 383 probands with lower and higher school scores, respectively. *P* values are two-tailed and uncorrected.

*LoFi* loss-of-function intolerant, *DD* developmental disorder, *OR* odds ratio, *CI* confidence interval.

A secondary analysis of their independent effects found both damaging de novo PTVs and damaging de novo missense variants contributed to school performance (Supplemental Table S4). All variants in the DD gene set were also in the LoFi gene set; after excluding variants in the DD gene set, we did not find significant support for association between damaging de novo coding variants in LoFi genes and lower school performance (uncorrected p value = 0.17; OR (95% CI) = 1.48 (0.85, 2.59)). Similar findings to those reported in Table 1 were observed when using an alternative definition of damaging coding variants (Supplementary Table S5).

No significant association with school performance was observed for transmitted or non-transmitted ultra-rare damaging coding variants, or for de novo, rare transmitted or rare non-transmitted CNVs (Table 1). We also found no significant associations when ultra-rare damaging coding variants and rare CNVs were analysed using a transmission disequilibrium test (Supplemental Tables S6 and S7). Moreover, no significant associations between CNVs (rare transmitted, rare non-transmitted or de novo) and school performance were observed when deletions and duplications were analysed separately (Supplemental Table S7).

### Phenotypic description of probands carrying damaging de novo variants in DD genes

A summary of phenotypes observed in the 13 carriers of damaging de novo coding variants in DD genes is provided in Supplemental Table S8. Among these probands, 7 obtained the lowest school score (grade = 3), 5 obtained the second lowest score (grade = 4), and one obtained the second from highest score (grade = 5). Thus, 54%, 38%, and 8% of probands with damaging de novo coding variants in DD genes had school grades of 3, 4 and 5, respectively. For comparison, 16%, 29%, and 30% of all sequenced probands had school grades of 3, 4 and 5, respectively.

A review of proband clinical notes indicated that among the 293 probands with lower school performance, the rate of comorbid mild ID in those who carried a damaging de novo coding variant in a DD gene (5 out of 12 probands) was significantly greater than the rate in those without a damaging de novo coding variant in a DD gene (12 out of 281 probands; Fisher’s exact P value = 2.2 × 10^–4^; OR (95% CI) = 15.6 (3.4, 67.7)). Additionally, among the 12 probands with lower school performance who also carried a damaging de novo coding variant in a DD gene, 9 developed schizophrenia after they had completed school (i.e. after the age of 18), indicating that these variants may have had premorbid effects on school performance.

### Multivariable analysis of rare and common variants

We performed a joint analysis of genetic factors that were significantly associated with school performance in the univariable analysis after correction for multiple testing. Damaging de novo coding variants in DD genes, PRS for EA and PRS for intelligence had significant independent effects on school performance (Table 2). The largest effect size and amount of variance explained were found for damaging de novo coding variants in DD genes (*β* = −2.64; Nagelkerke’s Δ*R*^2^ = 0.032), followed by PRS for intelligence (*β* = 0.27; Nagelkerke’s Δ*R*^2^ = 0.015) and PRS for EA (*β* = 0.23; Nagelkerke’s Δ*R*^2^ = 0.01) (Table 2). The total amount of variance explained by all genetic components associated with school performance was 5.7%. For completeness, we provide results from a joint analysis of all common and rare genetic factors analysed in our study in Supplementary Table S9.

**Table 2:** Multivariable logistic regression analysis of rare and common variants and school performance.

Genetic factor	Beta (95 % CIs)	*p* value	Variance explained (%)
Damaging de novo variants in DD genes	−2.64 (−4.9, −1.2)	1.3 × 10^–4^	3.24%
PRS educational attainment	0.23 (0.04, 0.42)	0.02	1.02%
PRS intelligence	0.27 (0.08, 0.46)	0.005	1.48%

Beta coefficients and *p* values were generated using a Firth’s logistic regression model. Variance explained were generated using binomial logistic regression models. The regressions adjusted for sex and 10 principal components and the binary school score dependent variable was coded as: 0 = lower school score, 1 = higher school score.

*DD* developmental disorder, *PRS* Polygenic Risk Score.

## Discussion

In an evaluation of the contribution from different classes of common and rare genetic variants, we found significant evidence for association between school performance in people with schizophrenia and PRS for EA and intelligence and damaging de novo variants in DD genes. Our results are broadly in line with previous findings that were derived from within-case, or population based, studies of cognitive function in schizophrenia [17, 19, 23]. However, while previous research reported only modest effects on cognition in schizophrenia from rare coding variants in LoFi genes or lists of DD genes [17, 22, 23], our trio-based design allowed us to identify de novo coding variants in DD genes that confer strong effects for lower school performance, and by implication cognitive impairment, in schizophrenia.

Probands in our study with lower school performance were enriched for damaging de novo coding variants in both LoFi genes (OR 2.07; 95% CI: 1.3, 3.5) and DD genes (OR 11.6; 95% CI: 2.8, 107), although the former did not show any nominally significant effect after excluding variants in the DD genes. Among all 13 probands with damaging de novo coding variants in DD genes, 12 had lower school performance, which provides evidence that it may be possible to use sequencing data to identify rare coding variants with large effects on cognition in a subgroup of people with schizophrenia. Of the 12 probands with lower school scores who carried a damaging de novo coding variant in a DD gene, 9 developed schizophrenia after they had completed school, which suggests that the effects of these variants on school performance are likely to be premorbid. However, we note that our findings are based on a small number of mutation carriers, and that school performance is only a proxy for cognition, therefore additional work is needed in larger samples with more comprehensive measures of cognition.

Our findings supported our hypothesis that for damaging coding variants, the strongest effects on school performance in schizophrenia come from de novo mutations. Indeed, we found no significant evidence for association between damaging ultra-rare transmitted coding variants and school performance. This is perhaps surprising given previous findings from within-case studies of cognition in schizophrenia [17, 23]. However, in our study, the proportion of mutations that were de novo among all ultra-rare damaging variants (i.e. de novo plus inherited variants) within LoFi genes and DD genes was 6% and 5%, respectively. Therefore, previous estimates of the effects from rare coding variants on cognition in schizophrenia are likely to be primarily based on inherited variants. This may explain the modest effect sizes for rare coding variants on cognition in schizophrenia reported in previous studies [17, 23], since we find significant effects only for de novo damaging coding variants. Different definitions of damaging coding variants may also contribute to differences in effect size that are reported in the current and previous studies. We also found no significant association between rare CNVs affecting LoFi genes or DD genes and school performance; given previous evidence for association between pathogenic CNVs and cognition in both schizophrenia and the general population [14, 43, 44], the most likely explanation is our study was underpowered to detect these effects.

The findings from our analysis of common variants are in line with those previously reported [17, 19] from case-control studies, where significant effects were found for intelligence and EA PRS on school performance, while no significant effect was found for schizophrenia PRS. We also reinforce these findings using the family structure of our sample, showing a greater overtransmission of intelligence and EA PRS to probands with higher school performance compared to probands with lower school performance.

Finally, within the context of the polygenic analysis, we performed the first investigation of the effects from parental non-transmitted alleles to school performance in schizophrenia, where we hypothesised that non-transmitted alleles for EA and intelligence would be associated with proband’s school performance. Contrary to our expectation, we found no significant evidence of genetic nurture from intelligence and EA PRS. This finding conflicts with previous research on EA within the general population [25], suggesting that genetic nurture effects might contribute less to EA in people with schizophrenia. However, in the general population, the indirect effects from genetic nurture on educational outcomes have been reported to be smaller than the direct effects from transmitted alleles [24]. Therefore, the amount of variance in school performance that is explained by non-transmitted PRS for cognitive traits in our sample is likely to be small. Given we had ~80% power at an alpha of 0.05 to detect PRS associations that explain ~1.8% of the variance in school performance, our study could be underpowered to detect small effects from non-transmitted parental alleles on school performance in schizophrenia.

In our multivariable model, de novo damaging coding variants in DD genes explained more variance in school performance (3.2%) than the combined effects from intelligence and EA PRS (2.5%). A previous within-case study [23, 24] reported ultra-rare PTVs and missense variants with MPC scores > 2 in LoFi genes explained a much smaller proportion of the variance for premorbid IQ (0.1%) and current cognition (0.2%), which underscores the importance of refining analytic approaches to prioritise pathogenic alleles, as we have done by focussing on de novo variants affecting known DD risk genes.

In this study, we used school performance as a proxy for cognitive ability. EA is often used as a substitute for cognition in genetic studies [17, 39, 45], as it is strongly associated with intelligence [46] and is more easily and more widely measured. However, school performance is not per se a cognitive phenotype and therefore its use comes with several limitations. First, we were unable to control for non-cognitive factors that affect school performance, for example socioeconomic status, educational opportunity, and both personal and environmental factors that influence motivation of an individual to perform well at school. Secondly, the absence of a direct measure of cognitive ability means we can only infer effects on cognition, and are unable to examine specific subdomains of cognition. Thirdly, mainstream schools in Bulgaria exclude people with severe or moderate ID, and our sample only included cases who completed mainstream school. Therefore, our study is underrepresented for people with schizophrenia who have the most severe forms of cognitive impairment. Although this ascertainment bias will not lead to false positive findings, it is likely to decrease power, particularly power to detect effects of rare mutations that are known to be enriched in people with schizophrenia and severe cognitive impairment, while also impacting the generalisability of our findings. Finally, we were not able to test whether the types of de novo variants we found to be associated with school performance have effects on cognition that only become manifest after the onset of the disorder. Despite these limitations, our study was able to provide insights into the genetic underpinnings of cognition in schizophrenia. Future studies should increase sample size, within the context of affected trios, as well as provide comparable samples of unaffected trios, which would be valuable for investigating possible differences in the genetic architecture of cognition in people with schizophrenia compared with the rest of the population. Our study also had the advantage of being based on a single, relatively homogeneous sample with a wealth of genetic data.

In summary, we aimed to investigate the genetic contribution to cognition in schizophrenia using school performance as a proxy. We found parental transmission of common genetic liability for EA and intelligence have direct effects on proband school performance, but non-transmitted parental alleles for EA and intelligence did not have indirect effects on proband school performance. Damaging de novo coding variants, but not damaging ultra-rare transmitted variants, within known DD genes have strong effects on school performance. Although carriers of damaging de novo coding variants in DD genes were enriched for mild ID, these mutations were not confined to people who had schizophrenia and comorbid ID. Instead, our findings provide novel evidence that some forms of schizophrenia are neurodevelopmental in origin [47], and that cognitive impairment in schizophrenia has a shared aetiology with DDs.

### Supplementary information

Supplementary material

## Data Availability

Exome sequencing data have previously been deposited in dbGaP: accession no. phs000687. Genotyping array data have been deposited in the PGC database, and a request to access these array data (dataset ID: ms.scz_xbutr_eur_sr-qc) can be made via the PGC data access portal (see https://pgc.unc.edu/for-researchers/data-access-committee/data-access-information/).

## Code availability

All code used for the analyses in this manuscript are available at https://git.cardiff.ac.uk/mpmar1/Family_based_analysis_of_rare_and_common_genetic_school_performance_schizophrenia.
