# Supplementary material for: Family-based analysis of the contribution of rare and common genetic variants to school performance in schizophrenia
Source: Mol Psychiatry. 2023 Mar 13;28(5):2081–7. doi: 10.1038/s41380-023-02013-2 (PMC10575776; doi:10.1038/s41380-023-02013-2)
Supplement: Supplementary file 1 — Supplementary material [file 41380_2023_2013_MOESM1_ESM.docx]

Supplemental Material for “Family-based analysis of the contribution of rare and common genetic variants to school performance in schizophrenia”

Contents

[Ethics committees that approved study protocol and recruitment 2](#_Toc124791137)

[Supplemental Figure S1: Distribution of school grades among probands included in the current study. 3](#_Toc124791138)

[Supplemental Figure S2. Principal Component Analysis Plot 4](#_Toc124791139)

[Supplemental Table S1: PRS results for different p-value thresholds in the source GWAS. 5](#_Toc124791140)

[Supplemental Table S2: pTDT results for different p-value thresholds in the source GWAS. 6](#_Toc124791141)

[Supplemental Table S3: Univariable logistic regression of mean parental PRS by proband school performance 7](#_Toc124791142)

[Supplemental Table S4: Association between different classes of de novo coding variant and school performance 8](#_Toc124791143)

[Supplemental Table S5: Analysis of an alternative definition of damaging coding variants and school performance. 9](#_Toc124791144)

[Supplemental Table S6: Transmission disequilibrium test of ultra-rare coding variants. 10](#_Toc124791145)

[Supplemental Table S7: Transmission disequilibrium test of rare CNVs 11](#_Toc124791146)

[Supplemental Table S8: Phenotypic description of probands carrying damaging *de novo* coding variants in developmental disorder genes 12](#_Toc124791147)

[Supplemental Table S9: Multivariable logistic regression analysis of all common variant polygenic risk scores and rare variants affecting developmental disorder genes 13](#_Toc124791148)

[Supplemental Figure S3: Univariable analysis of PRS between lower and higher school performance probands and their parents 14](#_Toc124791149)

# Ethics committees that approved study protocol and recruitment

Ethics Commission, Higher Medical University, **Plovdiv**, 4002 V Aprilov blvd 15a. Protocol N13/99

Ethics Committee to the Alexander University Hospital, **Sofia** 1431, 1 St G Sofiisk St, Protocol N71

Local Ethics Committee, District Dispensary for psychiatric disorders, **Russe**, bul Tutrakan 20, Protocol N5, 2002.

Ethics Committee at the State Psychiatric Hospital “Dr Georgi Kisiov”, **Radnevo**, 6269, Magda Petkanova St 1, Radnevo. Protocol of 2.10.2000.

Ethics Committee at the District Dispensary for psychiatric disorders, **Blagoevgrad**, Protocol N2/2000.

In the UK the project was approved by the Bro Taf Local Research Ethics Committee, Churchill House, 17 Churchill Way, Cardiff CF10 2TW, protocol 02/4523

# Supplemental Figure S1: Distribution of school grades among probands included in the current study.


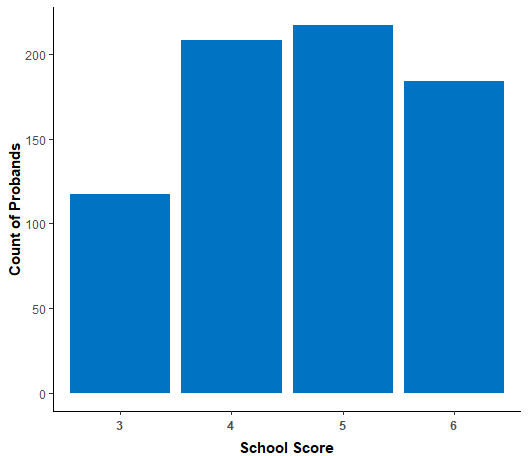


School grades ranged between 6 (the best possible grade) and 3 (the lowest passing grade). In the Bulgarian high school system, a grade of 2 is considered a failing grade, and students who fail are required to repeat their final school year until they receive a grade of at least 3. The probands included in our study were required to have completed mainstream school, and therefore all probands have a school grade of at least 3

# Supplemental Figure S2. Principal Component Analysis Plot


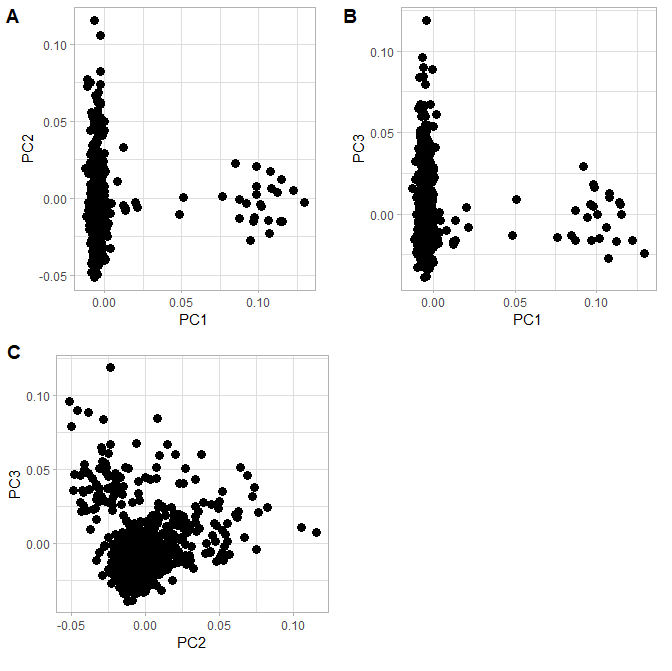


# Supplemental Table S1: PRS results for different p-value thresholds in the source GWAS.

| GWAS | Type | P-value Threshold | OR | OR lower 95% CI | OR upper 95% CI | p-value | % Variance Explained |
| --- | --- | --- | --- | --- | --- | --- | --- |
| EA | Proband PRS | 0.001 | 1.26 | 1.07 | 1.49 | 0.005 | 1.43 |
|  |  | 0.05 | 1.37 | 1.17 | 1.62 | 0.00015 | 2.63 |
|  |  | 0.5 | 1.36 | 1.15 | 1.60 | 0.00026 | 2.43 |
|  | ntPRS | 0.001 | 1.10 | 0.94 | 1.30 | 0.23 | 0.26 |
|  |  | 0.05 | 1.07 | 0.91 | 1.25 | 0.42 | 0.12 |
|  |  | 0.5 | 1.06 | 0.90 | 1.24 | 0.48 | 0.09 |
| SZ | Proband PRS | 0.001 | 0.99 | 0.85 | 1.17 | 0.93 | 0 |
|  |  | 0.05 | 1.01 | 0.86 | 1.18 | 0.94 | 0 |
|  |  | 0.5 | 1.07 | 0.91 | 1.26 | 0.42 | 0.12 |
|  | ntPRS | 0.001 | 0.94 | 0.80 | 1.10 | 0.43 | 0.11 |
|  |  | 0.05 | 1.00 | 0.85 | 1.17 | 0.98 | 0 |
|  |  | 0.5 | 0.96 | 0.81 | 1.13 | 0.61 | 0.05 |
| IQ | Proband PRS | 0.001 | 1.18 | 1.01 | 1.39 | 0.04 | 0.78 |
|  |  | 0.05 | 1.39 | 1.18 | 1.64 | 0.00009 | 2.80 |
|  |  | 0.5 | 1.39 | 1.18 | 1.64 | 0.00007 | 2.88 |
|  | ntPRS | 0.001 | 1.18 | 1.00 | 1.38 | 0.045 | 0.71 |
|  |  | 0.05 | 1.10 | 0.94 | 1.30 | 0.23 | 0.26 |
|  |  | 0.5 | 1.08 | 0.92 | 1.27 | 0.34 | 0.16 |

Logistic regression analysis where low school score probands are coded as 0, and high school score probands are coded as 1. Regression analysis controls for sex and principal components 1-10. P-values are uncorrected. Threshold = p-value threshold of the polygenic score. PRS = polygenic risk score; ntPRS = non-transmitted Polygenic Risk Score; EA = Educational Attainment; SZ = Schizophrenia; IQ = Intelligence; OR = Odds Ratio.

# Supplemental Table S2: pTDT results for different p-value thresholds in the source GWAS.

| PRS source GWAS | P-value Threshold | OR | OR lower 95% CI | OR upper 95% CI | p-value | % Variance Explained |
| --- | --- | --- | --- | --- | --- | --- |
| EA | 0.001 | 1.06 | 0.91 | 1.24 | 0.43 | 0 |
|  | 0.05 | 1.18 | 1.01 | 1.39 | **0.04** | 0.65 |
|  | 0.5 | 1.19 | 1.01 | 1.41 | **0.03** | 0.72 |
| SZ | 0.001 | 1.06 | 0.90 | 1.24 | 0.51 | 0 |
|  | 0.05 | 1.02 | 0.87 | 1.19 | 0.82 | 0 |
|  | 0.5 | 1.11 | 0.94 | 1.32 | 0.21 | 0.16 |
| IQ | 0.001 | 0.97 | 0.84 | 1.14 | 0.74 | 0 |
|  | 0.05 | 1.15 | 0.98 | 1.36 | 0.10 | 0.39 |
|  | 0.5 | 1.19 | 1.01 | 1.41 | **0.04** | 0.67 |

Logistic regression analysis where low school score probands are coded as 0, and high school score probands are coded as 1. Regression analysis controls for sex and principal components 1-10. P-values are uncorrected. Threshold = p-value threshold of the polygenic risk score. pTDT = polygenic transmission disequilibrium test; EA = Educational Attainment; SZ = Schizophrenia; IQ = Intelligence; OR = Odds Ratio

# Supplemental Table S3: Univariable logistic regression of mean parental PRS by proband school performance

| GWAS | P-value Threshold | OR (95% CI) | p-value | % Variance Explained |
| --- | --- | --- | --- | --- |
| EA | 0.001 | 1.28 (1.08, 1.51) | **0.004** | 1.49 |
|  | 0.05 | 1.31 (1.11, 1.55) | **0.0015** | 1.83 |
|  | 0.5 | 1.29 (1.09, 1.53) | **0.003** | 1.64 |
| SZ | 0.001 | 0.95 (0.81, 1.12) | 0.54 | 0 |
|  | 0.05 | 1 (0.85, 1.18) | 0.97 | 0 |
|  | 0.5 | 1.02 (0.86, 1.21) | 0.83 | 0 |
| IQ | 0.001 | 1.27 (1.08, 1.49) | **0.0038** | 1.50 |
|  | 0.05 | 1.35 (1.15, 1.6) | **0.0004** | 2.31 |
|  | 0.5 | 1.33 (1.13, 1.57) | **0.0006** | 2.11 |

Logistic regression analysis comparing mean parental PRS between probands with lower and higher school performance. Here, lower school score probands are coded as 0, and higher school score probands are coded as 1. Regression analysis controls for sex and principal components 1-10. P-values are uncorrected. Threshold = p-value threshold of the polygenic risk score. EA = educational attainment; SZ = schizophrenia; IQ = intelligence; OR = odds ratio; CI = confidence interval.

# Supplemental Table S4: Association between different classes of de novo coding variant and school performance

| **Gene set** | **Variant class** | **Odds ratio (95% CI)** | **P** | **N variants** | | **Average number of variants carried by probands** | |
| --- | --- | --- | --- | --- | --- | --- | --- |
|  |  |  |  | **Lower school scores** | **Higher school scores** | **Lower school scores** | **Higher school scores** |
| LoF intolerant genes | Damaging PTVs | 2.27 (1.05, 5.15) | 0.038 | 17 | 10 | 0.058 | 0.027 |
|  | Damaging missense variants | 1.86 (0.988, 3.62) | 0.0547 | 23 | 16 | 0.078 | 0.043 |
|  | Synonymous variants | 1.2 (0.571, 2.53) | 0.628 | 13 | 14 | 0.044 | 0.038 |
|  | Damaging coding variants | 2.07 (1.25, 3.5) | 0.00454 | 40 | 26 | 0.14 | 0.07 |
| DD genes | Damaging PTVs | 19.1 (2.21, 2500) | 0.00373 | 6 | 0 | 0.02 | 0 |
|  | Damaging missense variants | 5.5 (1.13, 53.4) | 0.0333 | 6 | 1 | 0.02 | 0.0027 |
|  | Synonymous variants | 0.962 (0.296, 2.94) | 0.946 | 5 | 7 | 0.017 | 0.019 |
|  | Damaging coding variants | 11.6 (2.74, 107) | 0.000263 | 12 | 1 | 0.041 | 0.0027 |

Firth’s penalised logistic regression analysis comparing the burden of coding *de novo* variants between probands with higher and lower school performance. Probands with lower and higher school performance are coded as 1 and 0 in the regression models, respectively. Regression analysis controls for sex and principal components 1-10. P-values are uncorrected The sequencing analysis of coding variants involved 293 and 369 probands with low and high school scores, respectively.

# Supplemental Table S5: Analysis of an alternative definition of damaging coding variants and school performance.

| Inheritance type | Gene set | OR (95% CI) | P | Variants carried by probands | |
| --- | --- | --- | --- | --- | --- |
|  |  |  |  | **Lower school scores** | **Higher school scores** |
| *De novo* | LoFi | 2.02 (1.08, 3.87) | 0.0285 | 25 | 17 |
|  | DD | 9.49 (2.15, 89.3) | 0.00173 | 9 | 1 |
| Transmitted | LoFi | 1.08 (0.881, 1.33) | 0.455 | 140 | 171 |
|  | DD | 0.964 (0.642, 1.42) | 0.854 | 36 | 44 |
| Non-transmitted | LoFi | 1.01 (0.815, 1.24) | 0.937 | 130 | 173 |
|  | DD | 0.754 (0.484, 1.16) | 0.199 | 35 | 61 |

In our previous publication (Creeth et al 2022), we defined damaging coding variants as Frameshift variants, stop-gain variants, splice donor/acceptor variants and missense variants with MPC scores ≥ 2, which differs to the criteria used in the current study (see methods in main text). Although fewer damaging variants are observed when using the Creeth et al definition of damaging coding variants, the findings are consistent with those reported in our primary analysis, where damaging coding de novo variants, but not damaging ultra-rare transmitted variants, in DD genes are associated with lower school performance. LoFi = loss-of-function intolerant. DD = developmental disorder.

# Supplemental Table S6: Transmission disequilibrium test of ultra-rare coding variants.

| **Gene set** | **Variant** | **School grade** | **Transmissions** | **Non-transmissions** | **Rate ratio** | **P-value** |
| --- | --- | --- | --- | --- | --- | --- |
| LoF intolerant genes | Damaging PTVs | Low | 50 | 48 | 1.04 | 0.84 |
|  |  | High | 57 | 62 | 0.919 | 0.65 |
|  | Damaging missense variants | Low | 397 | 371 | 1.07 | 0.35 |
|  |  | High | 525 | 491 | 1.07 | 0.29 |
|  | Damaging PTVs and missense variants | Low | 447 | 419 | 1.07 | 0.34 |
|  |  | High | 582 | 553 | 1.05 | 0.39 |
| DD genes | Damaging PTVs | Low | 13 | 10 | 1.3 | 0.53 |
|  |  | High | 12 | 20 | 0.6 | 0.16 |
|  | Damaging missense variants | Low | 101 | 84 | 1.2 | 0.21 |
|  |  | High | 140 | 130 | 1.08 | 0.54 |
|  | Damaging PTVs and missense variants | Low | 114 | 94 | 1.21 | 0.17 |
|  |  | High | 152 | 150 | 1.01 | 0.91 |

Transmission disequilibrium test of ultra-rare coding variants. The sequencing analysis of coding variants involved 293 and 369 probands with low and high school scores, respectively. P-values are uncorrected.

# Supplemental Table S7: Transmission disequilibrium test of rare CNVs

| **Gene set** | **School grade** | **CNV Type** | **Transmitted** | **Non-Transmitted** | **Rate ratio** | **p-value** |
| --- | --- | --- | --- | --- | --- | --- |
| LoF intolerant genes | Low | All | 40 | 39 | 1.02 | 0.9 |
|  |  | Deletions | 11 | 6 | 1.83 | 0.22 |
|  |  | Duplications | 29 | 33 | 0.88 | 0.61 |
|  | High | All | 46 | 53 | 0.87 | 0.48 |
|  |  | Deletions | 10 | 14 | 0.7 | 0.4 |
|  |  | Duplications | 36 | 39 | 0.92 | 0.72 |
| DD genes | Low | All | 8 | 12 | 0.72 | 0.49 |
|  |  | Deletions | 2 | 2 | 1 | 1 |
|  |  | Duplications | 6 | 10 | 0.6 | 0.3 |
|  | High | All | 8 | 11 | 0.66 | 0.37 |
|  |  | Deletions | 4 | 4 | 1 | 1 |
|  |  | Duplications | 4 | 7 | 0.57 | 0.36 |

Transmission disequilibrium test of rare CNVs. The TDT analysis of CNVs involved 310 and 383 probands with low and high school scores, respectively.

# Supplemental Table S8: Phenotypic description of probands carrying damaging *de novo* coding variants in developmental disorder genes

See separate Excel document “Supplemental_Table_S8.xlsx”. Age of schizophrenia onset is coded as non-overlapping 5-year age ranges, to ensure individual samples are not identifiable. DD = developmental disorder.

# Supplemental Table S9: Multivariable logistic regression analysis of all common variant polygenic risk scores and rare variants affecting developmental disorder genes

| Genetic factor | Inheritance type | Beta (95% CI) | p-value | Variance Explained |
| --- | --- | --- | --- | --- |
| EA PRS | Transmitted | 0.21 (0.02, 0.4) | 0.03 | 0.8% |
|  | Non-transmitted | 0.09 (-0.09, 0.28) | 0.3 | 0.16% |
| Intelligence PRS | Transmitted | 0.3 (0.1, 0.49) | 0.03 | 1.7% |
|  | Non-transmitted | 0.08 (-0.1, 0.27) | 0.4 | 0.13% |
| Schizophrenia PRS | Transmitted | 0.04 (-0.13, 0.21) | 0.6 | <0.1% |
|  | Non-transmitted | 0.03 (-0.14, 0.2) | 0.7 | <0.1% |
| Damaging rare coding variants in DD genes | *De novo* | -2.69 (-4.9, -1.2) | 0.0001 | 2.9% |
|  | Transmitted | 0.02 (-0.21, 0.26) | 0.8 | <0.1% |
|  | Non-transmitted | 0.23 (-0.03, 0.51) | 0.08 | 0.6% |
| Rare CNVs in DD genes | *De novo* | 0.22 (-1.65, 2.6) | 0.8 | <0.1% |
|  | Transmitted | -0.58 (-1.64, 0.48) | 0.3 | 0.2% |
|  | Non-transmitted | -0.28 (-1.17, 0.6) | 0.5 | <0.1% |

Beta coefficients and p-values were generated using a Firth’s logistic regression model. Variance explained were generated using binomial logistic regression models. The regressions adjusted for sex and 10 principal components and the binary school score dependent variable was coded as: 0 = lower school score, 1 = higher school score. DD = developmental disorder; PRS = Polygenic Risk Score. P values are uncorrected.

# Supplemental Figure S3: Univariable analysis of PRS between lower and higher school performance probands and their parents


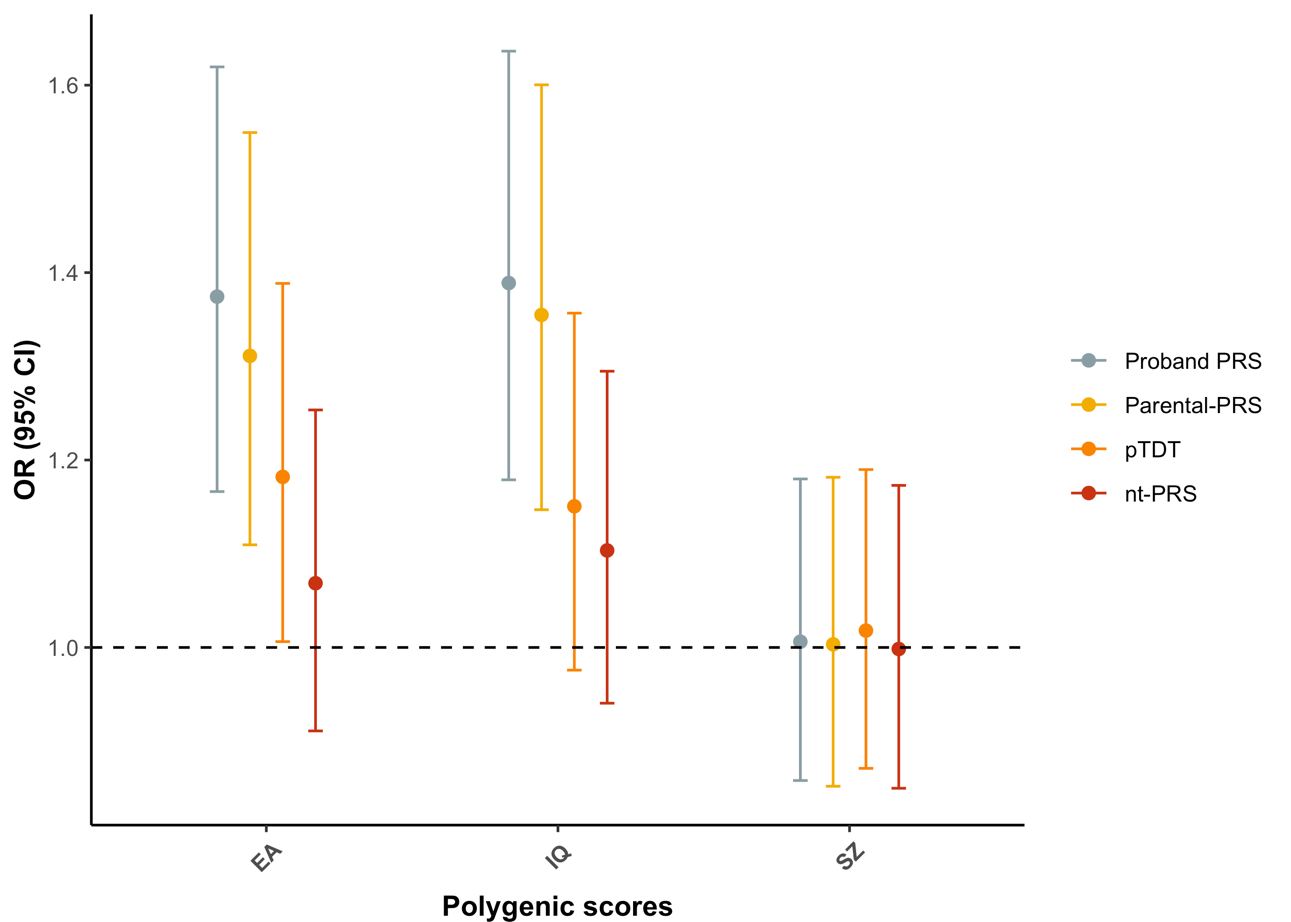


In the logistic regression model, the outcome variable is a binary measure of school performance, where probands with lower school scores are coded = 0, and probands with higher school scores are coded = 1. The regression models covary for proband sex + PC 1-10; PRS scores were generated from the source GWAS using a p-value threshold of 0.05.
